# Congenital CSF Rhinorrhoea sans Encephalocoele, sans Trauma, sans Tumour

**Published:** 2015-07-01

**Authors:** G Raghavendra Prasad, Kasha Aishwarya, Vijay sekhar

**Affiliations:** Deccan College of Medical Sciences, and Princess Esra Hospital, Hyderabad

**Keywords:** CSF rhinorrhoea, Cribriform defect, NeonateCongenital CSF rhinorrhoea

## Abstract

The authors describe a case of cerebrospinal fluid rhinorrhea due to a congenital defect in cribriform plate, an anomaly that has not been described hitherto. It was successfully treated surgically.

## INTRODUCTION

Cerebrospinal fluid (CSF) rhinorrhea is commonly reported with traumatic brain Injury, ruptured anterior encephalocele, brain tumors; idiopathic spontaneous CSF rhinorrhea is also known [1]. Spontaneous anterior fossa CSF rhinorrhoea and congenital perilymphatic fistula has been reported [2]. We report a neonate with normal intra-cranial pressure CSF rhinorrhea due to congenital defect in cribriform plate, and briefly describe the diagnostic difficulties and surgical management.

## CASE REPORT

A 27-day-oldneonate was brought with complaint of continuous watery discharge from left nostril from 2nd day of birth. He was a full term baby delivered at 36 weeks by LSCS with 9/10 APGAR score at 1min. There was no history of birth trauma, fever, seizures and other symptoms of raised intra-cranial tension or intracranial infection. The neonate was diagnosed as viral rhinitis by the pediatricians elsewhere and had been already administered 4 courses of anti-histaminics and antibiotics before he was brought to us in a dehydrated state. Examination of the baby was unremarkable without any external cranial trauma, any visible encephalocoele, normal inter-pupillary distance (if abnormal, it would have presence of ethmoidal encephalocele and normal neurological reflexes. The Reservoir test was positive, meaning the nasal discharge could be collected into a tube. The fluid examination values were consistent with that of CSF (glucose: 80 mg/dl, chlorides: 60 mEq/L, neutrophils: 6/phf). Beta-transferrin was positive confirming that the fluid was CSF. Cranial MRI revealed normal brain without any evidence of tumor or encephalocoele, but a continuous CSF flow in left nostril (Fig.1). 3D MR reconstruction of skull base revealed defect in the left cribriform plate (Fig. 1). Crista galli was deviated to the right. In view of the fact that there was no expertise to perform endoscopic trans-nasal repair and small endoscopes were unavailable in such a small baby, was contemplated but the senior author (GRP) decided to repair the defect through trans-frontal trans-dural approach. The defect was exposed by bi-temporal trans-frontal craniotomy. The skin flaps were raised up to the eyebrows. The dura was exposed by raising a laterally placed osteoblastic flap (Fig. 2a). It was opened with two dural tacker sutures in situ. The defect was exposed (Fig. 2b) and was plugged with abdominal wall fat, neuro-surgicel and a free peri-cranial graft (fig. 2c) Dura was closed continuous inter-locking sutures (Fig. 2d). The osteoblastic flap was restored followed by closure of skin. Instantaneous cessation of CSF leak was observed at the end of surgery (Fig. 2e). The post-operative period was uneventful. Follow up MRI after 9 months showed plug in place. (Fig. 2f). On 7 years follow-up, the child is thriving and growing well. 

**Figure:**
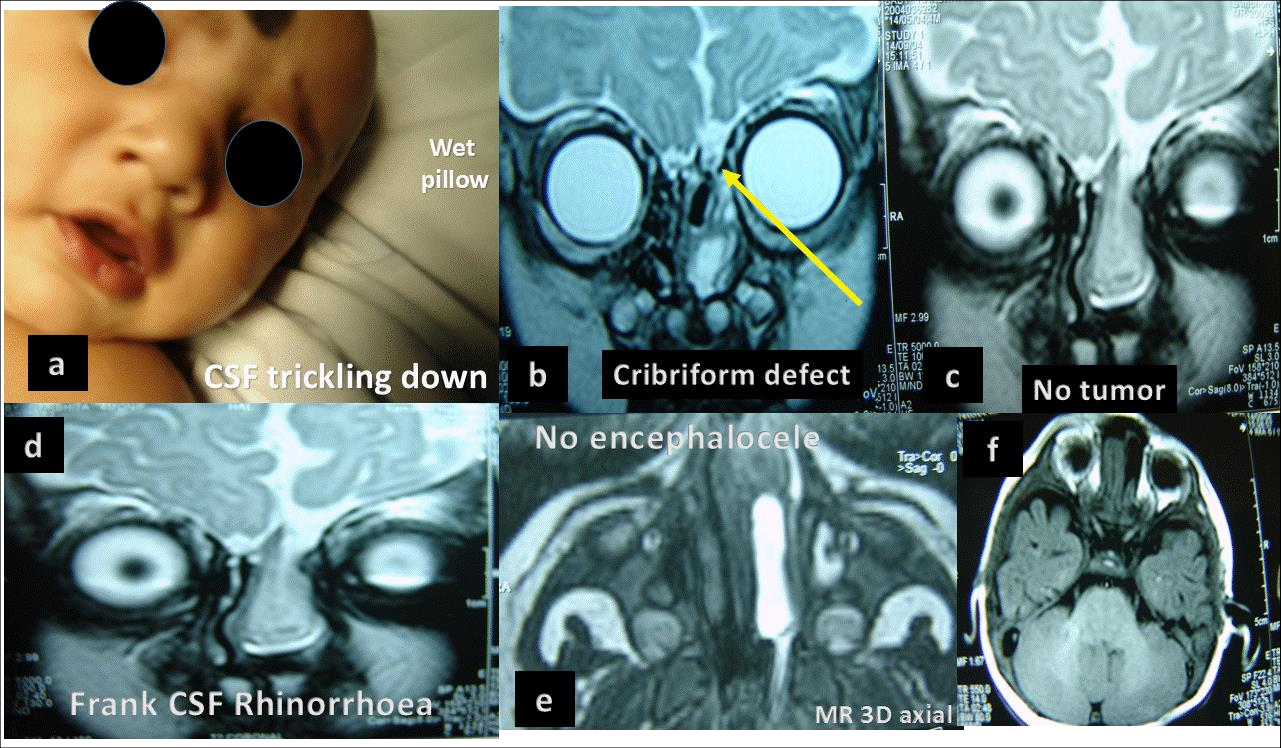
Fig. 1: a. Clinical picture showing CSF flowing out of nose b. MR Coronal showing cribriform defect c. MR showing no evidence of intracranial space occupying lesion. d. MR showing frank CSF rhinorrhea e. MR 3D axial showing no encephalocele f. MR Showing cranial base

**Figure:**
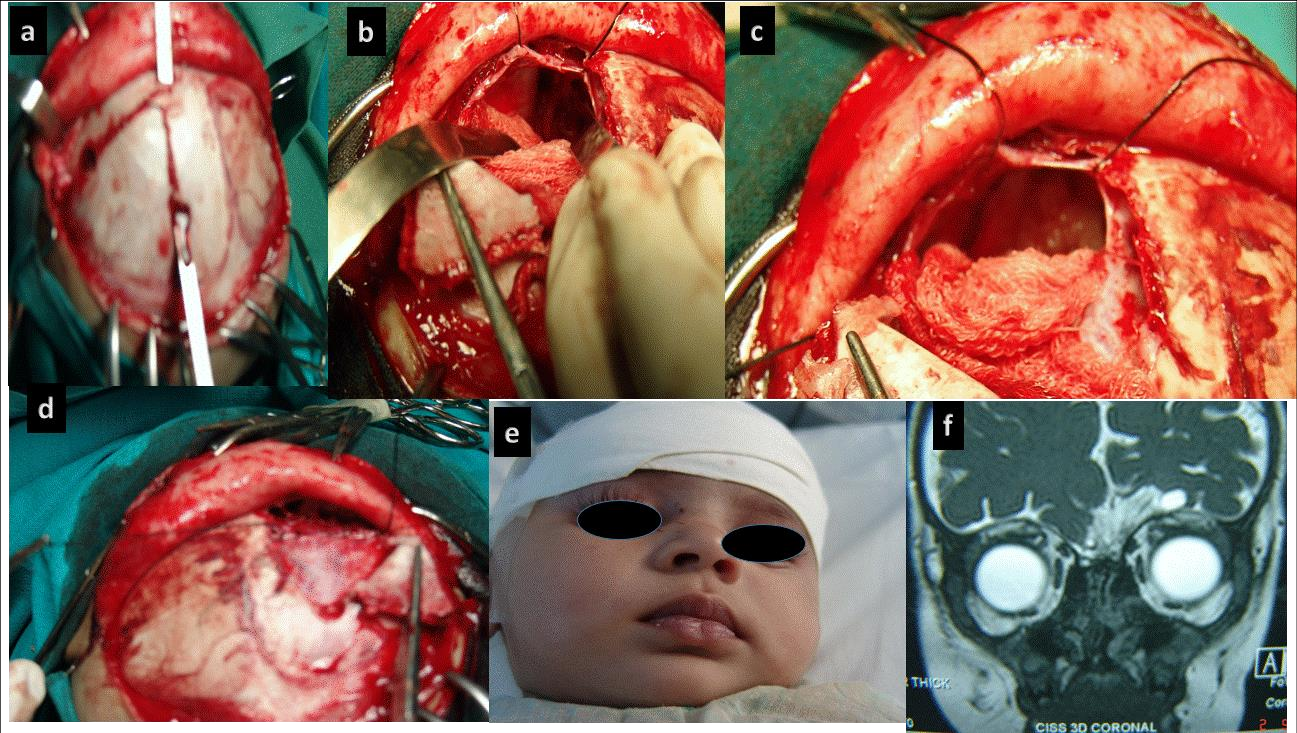
Fig. 2: a. Temporal based flap b. Defect in cribriform c. Fat plug in defect d. Dural closure e. Dry nose immediately at recovery f. Follow up MRI (9 months)

## DISCUSSION

Unilateral watery nasal discharge in an otherwise normal child should always be suspected to be CSF rhinorrhea. Unilateral, watery, clear rhinorrhea is extremely rare in viral rhinitis [3]. Unilateral nasal discharge in older children is a symptom qua-nun of foreign body nose. 

A reservoir test simply denotes the ability to collect the watery discharge from nose into a reservoir. This bedside test is not positive in other causes of nasal discharge. Although biochemical values had suggested CSF, beta-transferrin estimation, which is not universally available, is diagnostic of CSF [4]. MRI would clinch the diagnosis. 

Although a trial of conservative medical treatment is indicated in other causes of CSF rhinorrhea/ ottorrhea, CSF rhinorrhea with an anatomical cribriform defect will obviously not respond to conservative treatment and the child will be put to risk of meningitis. CSF rhinorrhea can be approached either trans-nasally, trans-sphenoidally or trans-frontally [5,6]. The open surgical approach has been already justified above.

## Footnotes

**Source of Support:** Nil

**Conflict of Interest:** Nil

